# Duplication with structural modification through extrachromosomal circular and lariat DNA in the human genome

**DOI:** 10.1038/s41598-020-63665-6

**Published:** 2020-04-28

**Authors:** Kazuki K. Takahashi, Hideki Innan

**Affiliations:** 1 SOKENDAI, The Graduate University for Advanced Studies, Hayama, Kanagawa 240–0193 Japan; 2 Laboratory of Plant Genetics, Graduate School of Agriculture, Kyoto University, Kyoto, 606–8502 Japan

**Keywords:** Evolutionary genetics, Mimicry

## Abstract

Duplication plays an important role in creating drastic changes in genome evolution. In addition to well-known tandem duplication, duplication can occur such that a duplicated DNA fragment is inserted at another location in the genome. Here, we report several genomic regions in the human genome that could be best explained by two types of insertion-based duplication mechanisms, where a duplicated DNA fragment was modified structurally and then inserted into the genome. In one process, the DNA fragment is turned into an extrachromosomal circular DNA, cut somewhere in the circle, and reintegrated into another location in the genome. And in the other, the DNA fragment forms a “lariat structure” with a “knot”, the strand is swapped at the knot, and is then reintegrated into the genome. Our results suggest that insertion-based duplication may not be a simple process; it may involve a complicated procedures such as structural modification before reintegration. However, the molecular mechanism has yet to be fully understood.

## Introduction

Various types of mutations can accumulate during genome evolution. Among these, duplication is thought to play an important role in providing drastic structural changes to the genome^1–3^. Segmental duplication accounts for up to 6% of the human genome^4^. Tandem duplication is the most well-known type of duplication and is generally initiated by non-allelic homologous recombination or by non-homologous end joining and replication-based mechanisms^5,6^. In addition, duplication can occur non-tandemly, that is, a duplicated region arises elsewhere in the genome. For this type of duplication, one may imagine that a duplicated fragment is inserted into an independent location some distance away from the corresponding segment, although the molecular mechanism not fully understood (see^6–8^).

Here, we report several interesting cases of insertion-based duplication in the human genome, where a duplicated DNA fragment is modified structurally, and then inserted into the genome. It has been reported that a DNA fragment can turn into extrachromosomal circular DNA (eccDNA) and can become reintegrated into the genome^9–11^. The existence of eccDNA in cancer cells (but not integrated in the nuclear genome) has been known for more than a half century^12,13^, but its presence in normal somatic cells has only been discovered recently^14^. It has also been reported that eccDNA is reintegrated back to the genome in cancer cells^9^, indicating that the reintegration of eccDNA may also occur in germ-line cells. Two clear demonstrations were reported in cattle^10^ and yeast^11^, and suggestive evidence was provided in human^15^. The findings in these cases provide fairly strong evidence for theories postulating the likelihood of eccDNA-mediated duplication in the human genome.

**Figure 1:**
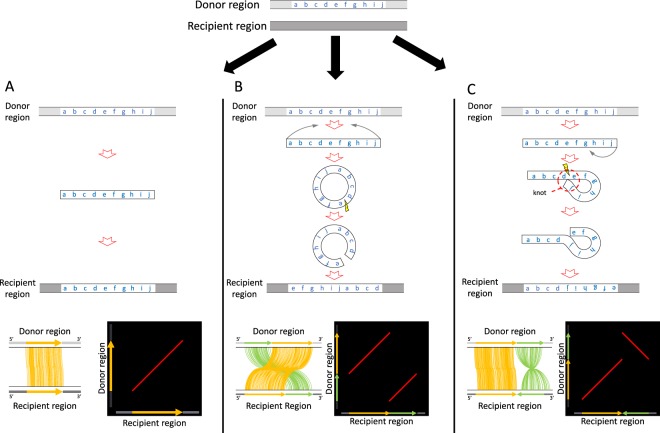
Illustration of eccDNA- and eltDNA-mediated duplications, compared to normal duplication with no modification involved. (**A**) Normal non-tandem duplication through insertion. The region from makers **a** to **j** is copied and inserted back to the recipient region in the genome, so that the paralogous regions keep the synteny as shown in the alignment, and a straight line arises in the dot plot of identity. (**B**) eccDNA-mediated duplication. The region from markers **a** to **j** is copied, turned into an eccDNA, and inserted back to the recipient region in the genome. Two parallel lines arise in the dot plot. (**C**) eltDNA-mediated duplication. The region from markers **a** to **j** is copied, turned into an eltDNA, the strand is swapped at the knot, and inserted back to the recipient region in the genome. Two orthogonal lines arise in the dot plot.

It is quite straightforward to detect eccDNA-mediated duplications in a genome, considering the process illustrated in Fig. 1B. Suppose that the region from markers **a** to **j** (donor region) is copied and turned into an eccDNA, cut somewhere in the circle (i.e., between markers **d** and **e**), and reintegrated into another independent location in the genome (recipient region). We could then observe a duplicated region in the syntenic order **efghijabcd** in the recipient region, which can be easily distinguished from the standard direct duplication that can be recognized as **abcdefghij** in the recipient region (Fig. 1A).

Furthermore, we report another type of duplication that may also involve structural modification before reintegration. We hypothesize that a DNA fragment could form a “lariat structure” (referred to as an extrachromosomal lariat DNA, or eltDNA), in which one end of the fragment is attached to the middle of itself (the attached point is referred to as a knot). Then, the strands are swapped at the knot, and it is reintegrate into the genome (see Fig. 1C). Suppose a knot was formed between markers **d** and **e**; we could observe a duplicated region in the syntenic order **abcdefghij**. We found a number of regions with strong evidence for eltDNA-mediated duplications in the human genome.

It seems that none of these observed duplications created new duplicated copies of coding genes and therefore may not directly contribute to adaptive genome evolution. However, it would be important to recognize that these kinds of structural modification processes may be involved when a region is duplicated. Such a change could potentially provide a selective advantage or disadvantage, for example, through expressional and/or epigenetical changes.

## Methods

We searched for eccDNA- and eltDNA-mediated duplications in the human genome. Segmental duplications (SDs) in the human genome were first comprehensively identified by Eichler and colleagues in GRCh35^16^. The authors defined SDs as duplicated regions with length >1 kb with nucleotide identity >90%.

Pu *et al*.^15^ updated the data of She *et al*.^16^ with the latest version, GRCh38, by developing the software SDquest. They identified 14,467 SDs (in total 198.3 Mb), which cover 95% (158.2 Mb) of the previously identified SDs by She *et al*.^16^ The sequence “homology” for most of them is >90%, but the data includes some SDs with 70–90% homology (see Table 1 in Pu *et al*.^15^), which seems lower compared to the results of She *et al*.^16^. The inconsistency could be due to the definition of homology; Pu *et al*.’s^15^ homology evaluates both nucleotide substitutions and indels. Throughout this article, we follow Pu *et al*.’s^15^ definition of homology, except when we construct NJ trees based on nucleotide substitutions.

**Figure 2:**
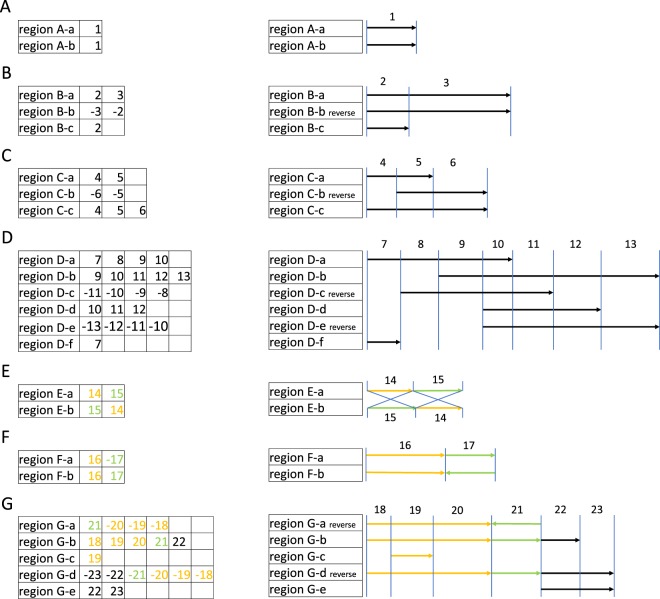
Grouping of Pu *et al*.’s^15^ eSD data. Examples are shown with hypothetical eSDs #1 to #23. See text for details.

Figure 2 explains the structure of Pu *et al*.’s^15^ data in the MosaicSDs_Human_hg38.txt file, which can be downloaded at https://github.com/SDquest/SDquest. Figure 2A describes the simplest type of SD with a pair of paralogous regions. An index number is given to the entire duplicated region ($\#$1 in this hypothetical example). A slightly complicated case, as illustrated in Fig. 2B, involves three regions (labeled B-a, -b, -c), two of which (B-a and -b) have homology in an extended region. In such a case, the entire region would be divided into two subregions named elementary SDs (eSDs) with an index number given to each eSD ($\#$2 and $\#$3 in Fig. 2B). The data can be tabulated as shown to the left of Fig. 2B. If homology is observed in the reverse strand, the index number is given as a negative value in the table (e.g., B-b in Fig. 2B, C-b in Fig. 2C). Figure 2D illustrates an example of complicated cases with many eSDs.

The data in MosaicSDs_Human_hg38.txt was grouped and tabulated, as illustrated in Fig. 2. By using the grouped eSD data, it was quite straightforward to search for eccDNA- and eltDNA-mediated duplications because they exhibit unique arrays of index numbers in the table. As shown in Fig. 2E, an eccDNA-mediated duplication should be detected as a pair of regions where the syntenic order of index numbers is different but the direction is the same (i.e., $\to$#14$\to$#15$\to$ vs. $\to$#15$\to$#14$\to$). An eltDNA-mediated duplication should be detected as a pair of regions with identical syntenic order of index numbers in the same direction in part, but in the opposite direction in the other (i.e., $\to$#16$\to$#17$\to$ vs. $\to$#16$\to$ $\leftarrow$#17$\leftarrow$) as show in the example of Fig. 2F. Figure 2G shows a complicated case that involves an eltDNA-mediated duplication (G-a) created from G-b. In addition, there are three regions (i.e., G-c, -d, and -e) exhibiting homology. In the following, when we find such a case, the full length regions of detected eltDNA- or eccDNA-mediated duplication will be shown (G-a, -b, and -d in the example of Fig. 2G, see below).

**Figure 3:**
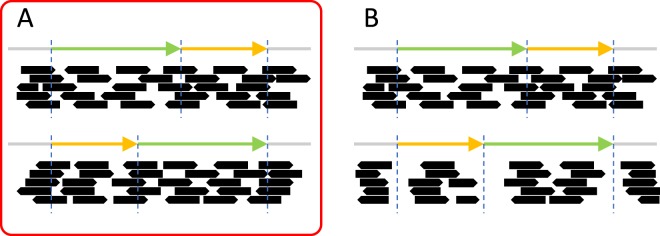
Illustration of predicted patterns of mapped NSG short-read data (**A**) when a detected eccDNA-mediated duplication is supported and (**B**) when not supported.

In order to confirm the presence of the detected eltDNA- or eccDNA-mediated duplications, we used NGS sequence data from the 1000 Genomes Project^17^. We arbitrarily chose 19 individuals representing the 19 “populations” defined in IGSR, The International Genome Sample Resource (see https://www.internationalgenome.org/faq/which-populations-are-part-your-study/). For these individual samples (summarized in Table S1), we downloaded high coverage whole genome sequence data from ftp://ftp.1000genomes.ebi.ac.uk, which were already mapped GRCh38 (hg38). For a structurally modified duplication, we predicted that both of the duplicated regions should be supported by the short-read data as illustrated in Fig. 3A (illustration for an eccDNA-mediated duplication), if they are really present in the genome. Alternatively, if it is an artifact, we should not be able to find reads supporting the breakpoints, as illustrated in Fig. 3B. As a result, we confirmed the presence of all detected eccDNA- and eltDNA-mediated duplications in all 19 individuals, indicating that they were not artifacts due to erroneous assembly of the reference genome.

For all identified eccDNA- and eltDNA-mediated duplications in the human genome, we checked whether their orthologous regions are present in chimpanzee and macaque genomes (panTro3 and rheMac8). For a first choice of outgroup species, we chose chimpanzee as the closest relative to us, which has quite a reliable reference genome. We did not use gorilla because the evolutionary distance from human is similar that for chimpanzee and the quality is not as good. Macaque and Orangutan were among our candidates for a second outgroup species, and we chose macaque because the quality of the macaque reference genome is much better than the orangutan one. By using to the synteny information of flanking coding regions in VGNG (https://vertebrate.genenames.org/), we narrowed down the orthologous regions, which were subject to detailed sequence-based analysis with BLAST 2.9.0^18^.

The codes used for our analyses are available at https://github.com/Kazuki526/circular_lariat.

## Results

We searched for eccDNA- and eltDNA-mediated duplications using data from Pu *et al*.^15^, following the strategy illustrated in Fig. 2. The data consisted of 15,312 regions with 14,467 distinct eSDs. We first grouped them such that all regions in the same group shared at least one eSDs (see Fig. 2). We obtained 3,924 of such groups (mean 3.9, median 2, standard deviation (SD) 62.15) The majority of the groups (99.3%) had <20 regions, with maximum 89, if one obvious outlier (with 3890 regions) was excluded.

For detecting eccDNA- and eltDNA-mediated duplications, we found, respectively, 52 and 77 regions that were consistent with the patterns illustrated in Fig. 2E,F. However, we found that these regions included a number of false positives, which were easily excluded by looking at dot plots of surrounding regions.

**Figure 4:**
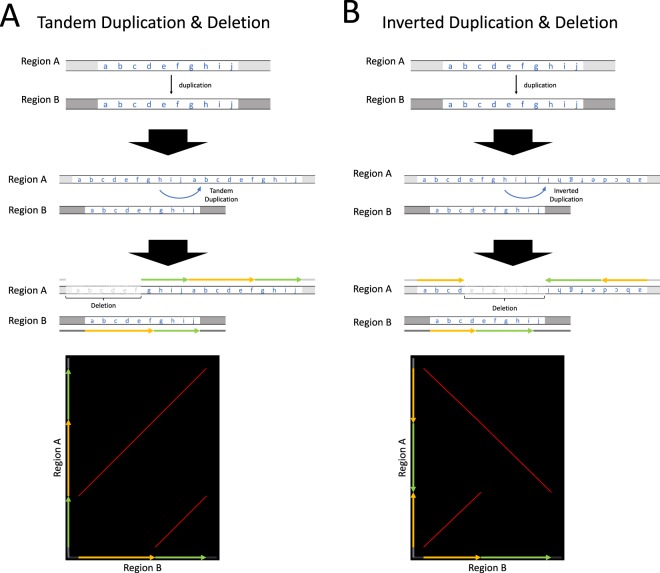
Typical examples of false positive signatures for (**A**) eccDNA- and (**B**) eltDNA-mediated duplications. (**A**) A tandem duplication in the donor region, a duplication to the recipient region, followed by a partial deletion in the donor region, could create a similar pattern to that predicted by an eccDNA-mediated duplication in the dot plot. (**B**) A head-to-head duplication in the donor region, a duplication to the recipient region, followed by a partial deletion in the donor region, could create a similar pattern to that predicted by eltDNA-mediated duplication in the dot plot. See text for details.

Typical examples of false positives are illustrated in Fig. 4. Figure 4A involves duplication events twice followed by a partial deletion. Region A (**abcdefghij**) is first duplicated into region B, and then region A undergoes a tandem duplication again, resulting in (**abcdefghijabcdefghij**). Next, a part of the tandemly duplicated region (**abcdef**) is deleted. Then, if we compare a part of region A (**ghijabcdef**) and region B (**abcdefghij**), the pattern is completely consistent with that expected by an eccDNA-mediated duplication (see Fig. 1B), that is, the order of the yellow and green regions (**abcdef** and **ghij**, respectively) is swapped. However, this pattern can be recognized as a false positive if we find that there are two green regions flanking the yellow region. Similarly, Fig. 4B shows a false positive pattern of an eltDNA-mediated duplication arising from head-to-head duplication, followed by duplication to an independent genomic location and a partial deletion. In all cases, if we look at a partial region, the pattern seems to be consistent with eccDNA- or eltDNA-mediated duplication, but not in the whole region. Although these patterns do not necessarily rule out the possibility of eccDNA- or eltDNA-mediated duplication, to be conservative, we excluded these cases. Then, we finally identified 3 and 20 fairly strong candidates for eccDNA- and eltDNA-mediated duplications (Table S2).

We checked whether these detected regions were present in the whole genome sequence data in 19 human individuals in the 1000 Genomes Project (see Method). We confirmed that all of them were present, by mapping next-generation short-read data with special attention to the regions encompassing the breakpoints that specify the synteny of eSDs involved in eccDNA- and eltDNA-mediated duplications. It is indicated that all detected eccDNA- and eltDNA-mediated duplications are not due to erroneous assembly of the reference genome.

We also used the chimpanzee and macaque genomes to investigated whether their orthologous regions were present (see Method). For each region of the identified eccDNA- and eltDNA-mediated duplications, presence/absence was examined in its 5′ and 3′ regions and in the orthologous part in the chimpanzee and macaque genomes. The result is summarized in Table 1, where $\circ$, ×, and Δ represent the presence, absence and partial presence of the region, respectively. For a pair of duplicated regions, the donor/recipient copies can be determined when we observe the pattern ($\circ$, $\circ$, $\circ$)/($\circ$, ×, $\circ$), where the three symbols in each pair of parenthesis exhibit the presence/absence of the 5′ flanking, the focal duplicated region, and the 3′ flanking regions (see Table 1 for details).

**Table 1:** Summary of coding overlap in the human genome, and presence/absence of the detected eccDNA- and eltDNA-mediated duplications in the chimpanzee and macaque genomes.

Region	Overlap with coding genes in the human genome			Presence/absence in Chimpanzee			Presence/absence in Macaque		
C1a	—	—	—	$\circ$	$\circ$	$\circ$	$\circ$	$\circ$	$\circ$
C1b	—	—	—	$\times$	$\times$	$\times$	_Δ_	$\times$	$\times$
C2a	—	TCAF1	TCAF1	$\times$	$\times$	$\times$	$\times$	$\times$	$\times$
C2b	—	TCAF2, TCAF2C	—	$\circ$	_Δ_	$\times$	$\times$	$\times$	$\times$
C3a	—	—	—	$\circ$	$\circ$	$\circ$	$\circ$	$\times$	$\circ$
C3b	—	—	—	$\times$	$\times$	$\times$	$\times$	$\times$	$\times$
C3c	—	—	—	$\times$	$\times$	$\times$	$\times$	$\times$	$\times$
C3d	—	—	—	$\times$	$\times$	$\times$	$\times$	$\times$	$\times$
C3e	—	—	—	$\times$	$\times$	$\times$	$\times$	$\times$	$\times$
C3f	—	—	—	$\times$	$\times$	$\times$	$\times$	$\times$	$\times$
C3g	—	—	—	$\circ$	$\circ$	$\circ$	$\times$	$\times$	$\times$
C3h	—	—	—	$\times$	$\times$	$\times$	$\times$	$\times$	$\times$
C3i	—	—	—	$\times$	$\times$	$\times$	$\times$	$\times$	$\times$
C3j	—	—	—	$\times$	$\times$	$\times$	$\times$	$\times$	$\times$
C3k	—	—	—	$\times$	$\times$	$\times$	$\times$	$\times$	$\times$
C3l	—	—	—	$\times$	$\times$	$\times$	$\times$	$\times$	$\times$
C3m	—	—	—	$\times$	$\times$	$\times$	$\times$	$\times$	$\times$
C3n	—	—	—	$\circ$	$\circ$	$\circ$	$\times$	$\times$	$\times$
C3o	PCMTD2	—	—	$\circ$	_Δ_	$\times$	$\times$	$\times$	$\times$
L1a	—	—	—	$\circ$	$\circ$	$\circ$	$\times$	$\times$	$\times$
L1b	ATM	ATM	ATM	$\circ$	$\circ$	$\circ$	$\circ$	$\circ$	$\circ$
L2a	—	—	—	$\circ$	$\circ$	$\circ$	$\circ$	$\times$	$\circ$
L2b	—	—	—	$\circ$	$\circ$	$\circ$	$\circ$	$\circ$	$\circ$
L2c	—	—	—	$\circ$	$\circ$	$\circ$	_Δ_	$\times$	_Δ_
L2d	—	—	—	$\circ$	$\circ$	$\circ$	$\circ$	$\circ$	$\circ$
L3a	—	—	—	$\circ$	$\circ$	$\circ$	$\circ$	$\circ$	$\circ$
L3b	—	—	—	$\circ$	$\circ$	$\circ$	$\circ$	$\circ$	$\circ$
L3c	—	—	—	$\circ$	$\circ$	$\circ$	$\circ$	$\circ$	$\circ$
L4a	—	—	—	$\circ$	$\circ$	$\circ$	_Δ_	$\times$	$\times$
L4b	—	—	UGT2A1,UGT2A2	$\circ$	$\circ$	$\circ$	$\circ$	$\circ$	$\circ$
L4c	ANKRD6, LYRM2	ANKRD6, LYRM2	ANKRD6, LYRM2, MDN1	$\times$	$\times$	$\times$	$\times$	$\times$	$\times$
L5a	—	—	—	$\circ$	$\circ$	$\circ$	$\circ$	$\circ$	$\circ$
L5b	—	—	—	$\circ$	$\circ$	$\circ$	$\circ$	$\circ$	$\circ$
L6a	—	—	—	$\circ$	$\circ$	$\circ$	$\circ$	$\circ$	$\circ$
L6b	—	—	—	$\circ$	$\circ$	$\circ$	$\circ$	$\circ$	$\circ$
L6c	ARHGAP5	ARHGAP5	ARHGAP5	$\circ$	$\circ$	$\circ$	$\circ$	$\circ$	$\circ$
L7a	—	—	—	$\circ$	$\circ$	$\circ$	$\circ$	$\circ$	$\circ$
L7b	—	—	—	$\circ$	$\circ$	$\circ$	$\times$	_Δ_	$\circ$
L7c	ERCC6	ERCC6	ERCC6	$\circ$	$\circ$	$\circ$	$\circ$	$\circ$	$\circ$
L7d	—	—	—	$\circ$	$\circ$	$\circ$	$\times$	_Δ_	$\circ$
L8a	—	—	—	$\circ$	$\circ$	$\circ$	$\circ$	$\circ$	$\circ$
L8b	—	—	—	$\circ$	$\circ$	$\circ$	$\circ$	$\circ$	$\circ$
L8c	DNM1L	—	DNM1L, YARS2	$\circ$	$\circ$	$\circ$	$\circ$	$\circ$	$\circ$
L8d	PHLDA1	NAP1L1	NAP1L1	$\circ$	$\circ$	$\circ$	$\circ$	$\circ$	$\circ$
L9a	—	—	—	$\circ$	$\circ$	$\circ$	$\circ$	$\circ$	$\circ$
L9b	THSD1	VPS36	VPS36	$\times$	$\times$	$\times$	$\circ$	$\circ$	$\circ$
L10a	—	—	—	$\circ$	$\circ$	$\circ$	$\times$	$\times$	$\times$
L10b	—	—	—	$\circ$	$\circ$	$\circ$	$\times$	$\times$	$\times$
L11a	—	—	—	$\circ$	$\circ$	$\circ$	$\circ$	$\circ$	$\circ$
L11b	—	—	—	$\circ$	$\circ$	$\circ$	$\circ$	_Δ_	$\times$
L12a	—	—	—	$\circ$	$\circ$	$\circ$	$\circ$	$\circ$	$\circ$
L12b	HAUS2	—	STARD9	$\circ$	$\circ$	$\circ$	$\circ$	$\circ$	$\circ$
L13a	—	—	—	$\circ$	$\circ$	$\circ$	$\circ$	$\circ$	_Δ_
L13b	ZDHHC20	ZDHHC20	ZDHHC20	$\circ$	$\circ$	$\circ$	_Δ_	$\circ$	$\circ$
L13c	—	—	—	$\circ$	$\circ$	$\circ$	$\circ$	$\circ$	$\circ$
L14a	—	—	—	$\circ$	$\circ$	$\circ$	$\circ$	$\circ$	$\circ$
L14b	MINDY2	MINDY2	MINDY2	$\times$	$\times$	$\times$	$\circ$	$\circ$	$\circ$
L15a	—	—	—	$\circ$	$\circ$	$\circ$	$\circ$	$\circ$	$\circ$
L15b	—	—	—	$\circ$	$\circ$	$\circ$	$\times$	$\times$	$\times$
L15c	—	—	—	$\circ$	$\circ$	$\circ$	$\times$	$\times$	$\times$
L16a	—	—	—	$\circ$	$\circ$	$\circ$	$\times$	$\times$	$\times$
L16b	—	—	—	$\circ$	$\circ$	$\circ$	$\times$	$\times$	$\times$
L17a	PRRC2C	PRRC2C	MYOCOS	$\circ$	$\circ$	$\circ$	$\circ$	$\circ$	_Δ_
L17b	—	—	—	$\circ$	$\circ$	$\circ$	$\circ$	$\circ$	$\circ$
L17c	—	—	—	$\times$	$\times$	$\times$	$\times$	$\times$	$\times$
L18a	—	—	PDS5A	$\circ$	$\circ$	$\circ$	$\times$	$\times$	$\times$
L18b	—	—	—	$\circ$	$\circ$	$\circ$	$\times$	$\times$	$\times$
L18c	PABPC3	PABPC3	PABPC3	$\circ$	$\circ$	$\circ$	$\times$	$\times$	$\times$
L19a	STK31	—	STK31	$\circ$	$\circ$	$\circ$	$\times$	$\times$	$\times$
L19b	PXDNL,PCMTD1	PCMTD1	—	$\circ$	$\circ$	$\circ$	$\times$	$\times$	$\times$
L19c	—	—	—	$\times$	$\times$	$\times$	$\times$	$\times$	$\times$
L19d	—	—	—	$\circ$	$\circ$	$\circ$	$\times$	$\times$	$\times$
L19e	—	—	—	$\circ$	$\circ$	$\circ$	$\times$	$\times$	$\times$
L19f	—	—	—	%	$\circ$	$\circ$	$\times$	$\times$	$\times$
L19g	—	—	—	$\times$	$\times$	$\times$	$\times$	$\times$	$\times$
L19h	—	—	—	$\times$	$\times$	$\times$	$\times$	$\times$	$\times$
L20a	—	—	—	–	$\circ$	$\circ$	$\circ$	$\circ$	$\circ$
L20a	—	—	—	$\circ$	$\circ$	$\circ$	$\times$	$\times$	$\times$

See text and Table S3 for details.

### eccDNA-mediated duplication

**Figure 5:**
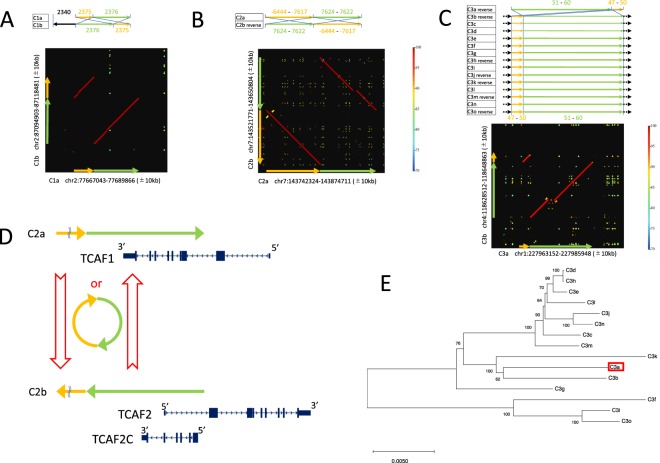
(**A**–**C**) Three candidate regions for eccDNA-mediated duplications. For each, an alignment of eSD# and a dot plot are shown. Dot-plots were produced by using GenomeMatcher with its default setting^19^ (**D**) Overlapped coding genes in C2. (**E**) Unrooted NJ tree of the 15 regions involved in C3, which identified the donor and recipient copies (C3b and C3a, respectively). The copy created by eccDNA-mediated duplication is boxed in red. The tree was made using MEGA X^20^. The bootstrap support in percentage is presented for internal branches.

We found three cases with fairly strong signatures of eccDNA-mediated duplications. Figure 5A is a simple case (C1), where two distinct regions (C1a and C1b, both ~23 kb in length) on chromosome 2 were involved. The duplicated regions consist of two eSDs ($\#$2375 in yellow and #2376 in green in Fig. 5A), and the sequence homology between the two paralogous regions is 97.0%. We found orthologous regions of C1a and C1b as ($\circ$, $\circ$, $\circ$) and ($\times$, $\times$, $\times$) in the chimpanzee genome, respectively, and ($\circ$, $\circ$, $\circ$) and (Δ, $\times$, $\times$) in the macaque genome, respectively. However, from this observed pattern, it was difficult to determine their donor/recipient statuses. We found that these regions had no overlap with coding genes, which did not help to determine the donor/recipient statuses (see below for a successful case).

Figure 5B is another case (C2) with two distinct regions in chromosome 7 (C2a and C2b, both ~130 kb in length). The sequence homology between the two paralogous regions is 95.3%. We found that the orthologous regions of C2a were absent in the chimpanzee and macaque genomes, while the orthologous region of C2b was partially found in the chimpanzee genome, making it difficult to determine the donor/recipient status. The duplicates show an interesting pattern of coding gene overlap, as illustrated in Fig. 5D. C2a overlaps with a 3′ half of TCAF1, while C2b overlaps with 5′UTR of TCAF2. Furthermore, TCAF2C resides completely within C2b, which cannot be considered a partial duplicate of TCAF1 because they are on the different strands. See the DISCUSSION for this complex pattern.

The third case (C3) involves 15 regions with length about ~20 kb, where the synteny of C3-a is different from that of the others (Fig. 5C). An NJ tree of the 15 regions is shown in Fig. 5E, indicating that C3a was recently derived through an eccDNA-mediated duplication from C3b. The sequence homology C3a and the closet copy C3b is 96.7%.

### eltDNA-mediated duplication

**Figure 6:**
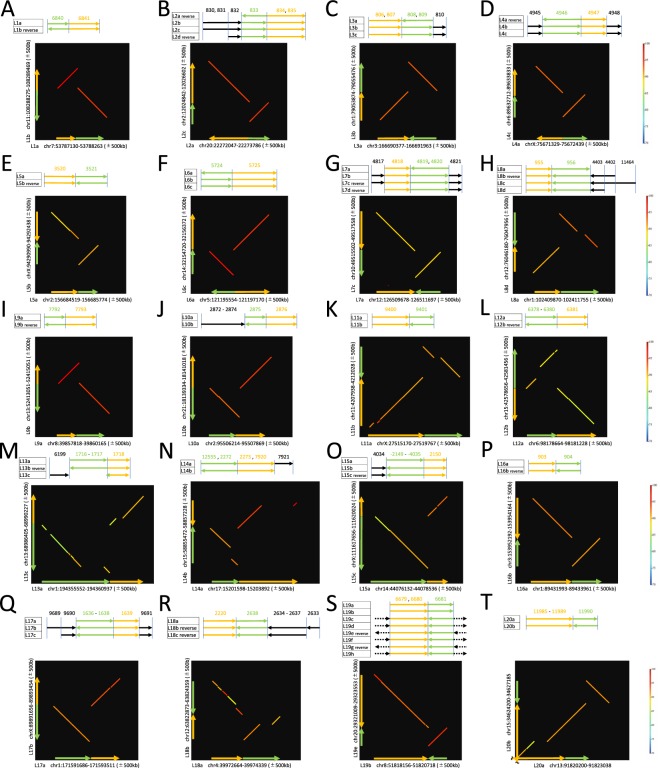
(**A**–**T**) 20 candidate regions of eltDNA-mediated duplications. For each, an alignment of eSD# and a dot plot are shown. For L20 (**T**), which is 26 kb long, only a part of the duplicated region is shown here. Dot-plots were produced by using GenomeMatcher with its default setting^19^.

**Figure 7:**
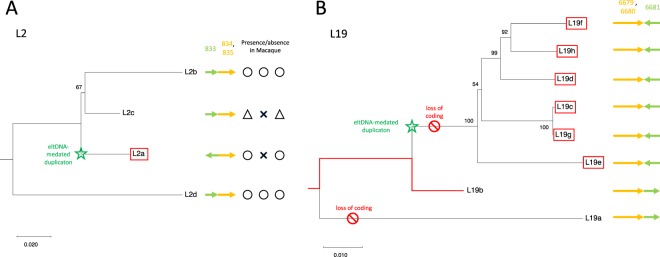
(**A**,**B**) NJ trees for L2 and L19. The trees were made by MEGA X^20^. The copy created by eltDNA-mediated duplication is boxed in red. The numbers on the tree are bootstrap values for internal lineages. The lineage is shown in red when the region overlaps with a coding gene.

We found 20 cases with strong signatures of eltDNA-mediated duplication (Fig. 6). The lengths of duplicated regions distribute in a relatively narrow range of around 2–3 kb (mean 2,854 bp, median 1,934 bp, SD 4258), and if an exceptionally long one (L20, 25 kb) is excluded, and the average homology is 85.8% (SD 5.58%). Table 1 summarizes the overlap with coding genes and the presence/absence statuses in the chimpanzee and macaque genomes. In 11 cases (L1, L4, L6, L7, L8, L9, L13, L14, L17, L18, L19), at most, one region in each case exhibited an overlap with a coding gene. If we assume that duplication of a noncoding region is unlikely to create a novel coding gene, we may be able to assume that the copy with coding gene overlapped should be the donor. This prediction seems to be correct at least for L19 (see Fig. 7B for an NJ tree), where L19b overlaped with PCMTD1 and all others seemed to have lost the function after duplication. L19-a seems to be the recipient, which was inserted in an intronic region of STK31 (see Table 1).

Overall, the presence/absence information in the chimpanzee and macaque genomes was not very informative mainly because in many cases, large regions encompassing the focal duplicate regions are missing (i.e., ($\times$, $\times$, $\times$)). The only an exception was L2, where a convincing pattern ($\circ$, $\times$, $\circ$) was observed for L2a in the macaque genome. It seems that there were two copies (L2b and L2d) in the genome of the ancestor of humans and macaques, and in the current macaque genome. After the split between humans and macaques, two duplications occurred in the lineage of humans to create L2a and L2b, one of which involved eltDNA-mediated modification (L2a). This is consistent with the NJ tree in Fig. 7A, which demonstrates L2a as a young copy, although the donor copy was not clearly determined due to a relatively low bootstrap value (66/100) for the internal branch.

## Discussion

**Figure 8:**
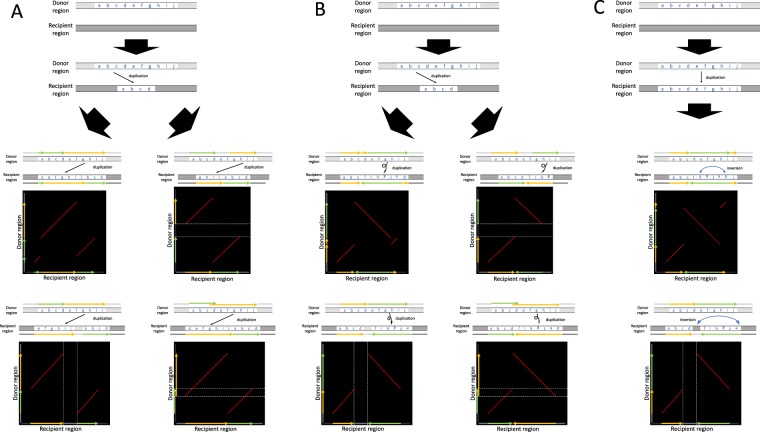
Illustration of possible patterns involving multiple duplications and inversions. (**A**) Those showing similar patterns to after an eccDNA-mediated duplication. (**B**,**C**) Those showing similar patterns to after an eltDNA-mediated duplication. In (**B**), two duplication events are involved, while in (**C**) a duplication and an inversion event are considered.

In this study, we found 3 and 20 cases with fairly strong evidence for eccDNA- and eltDNA-mediated duplications, respectively. We did not rule out other mutational scenarios that explain the detected regions. Indeed, one may think that the observed patterns may be explained by involving multiple duplication/deletion events. Nevertheless, we would emphasize that such a scenario would be quite unlikely, as illustrated in Fig. 8. Figure 8A examines whether two duplication events can explain an eccDNA-mediated duplication. Consider a donor region **abcdefghij** and suppose that a part of the region (**abcd**, presented by a yellow arrow) is first duplicated and inserted in the recipient region. If the remaining part (**efghij**, presented by a green arrow) is then duplicated and inserted exactly at the 5′ breakpoint of the former insertion, it could result in the pattern predicted by an eccDNA-mediated duplication (i.e., **efghijabcd**).

However, the odds that the secondary insertion comes at this exact breakpoint should be extremely low. If an insertion is randomly placed in the genome, the odds would be $1/(3\times {10}^{9})$ assuming the genome size to be $3\times {10}^{9}$. Although we did not find any evidence that insertion occurred with local homology, if an insertion requires a 6 bp match, the odds would be $1/\{2\times {(1/4)}^{6}\times 3\times {10}^{9}\}\simeq 6.8\times {10}^{-7}$ (assuming equal frequencies of four nucleotides). It should be noted that, if the secondary insertion comes slightly upstream or downstream, the outcome will be different from that of an eccDNA-mediated duplication, so that we can distinguish them. Figure 8A clearly demonstrates that such cases would be distinguished in dot plots, where there is a gap or an overlap between the yellow and green arrows. The same logic also applies to an eltDNA-mediated duplication. A duplication (**abcd**, yellow arrow) followed by an inverted duplication at the 3′ breakpoint of the former duplication could exhibit the same pattern as an eltDNA-mediated duplication, but we emphasize that this is only true when the secondary duplication is inserted at the 3′ breakpoint of the primary duplication (Fig. 8B). Similarly, it is difficult to explain by a duplication followed by an inversion as shown in Fig. 8C. Thus, the likelihood that multiple duplication/deletion/inversion events explain the 23 identified duplicated regions should be low.

A caveat applies to the two cases, C1 and C2, where the duplicates are located on the same chromosome with 10 Mb and 200 kb intervals, respectively. For these cases, complex nested duplications and structural changes may have created the observed patterns. Indeed, there are many copies of the same eSDs in the surrounding region of C2. There also are multiple copies of the TCAF gene family, which could explain the unique observation that both donor and recipient regions overlap with coding genes. Nevertheless, we demonstrate that duplication involving structural modification should play role in the human genome evolution.

It is found that all identified cases have flipped regions with length more than 500 bp, which is identical to the minimum length of eSDs^15^. It is indicated that there may be a number of undetected eccDNA- and eltDNA-mediated duplications in the human genome, most likely smaller than 500 bp long.

The presence of eccDNAs that are not integrated in the host chromosome is well-known, particularly in cancer cells, and there is some evidence that eccDNA can be reintegrated into the genome in cattle^10^ and yeast^11^. We have demonstrated that reintegration of eccDNA could occur in the human genome, indicating that duplication may occasionally involve structural modifications before reintegration, rather than simply inserting a copied region. An eltDNA could be another type of such structural modification. Our results suggest that insertion-based duplication may not be a simple process; it may involve a complicated process such as structural modification before reintegration, although the molecular mechanism is not yet fully understood. Such modifications may potentially contribute to adaptive genome evolution, although we did not find any clear evidence for this in our data.

## Supplementary information

Supplementary Information.

Supplementary Information2.

Supplementary Information3.
